# Supplementary material for: Ancestral and recent bursts of transposition shaped the massive genomes of plant pathogenic rust fungi
Source: BMC Genomics. 2025 Jul 1;26:627. doi: 10.1186/s12864-025-11726-3 (PMC12210899; doi:10.1186/s12864-025-11726-3)
Supplement: Supplementary file 1 — Supplementary Material 1: Fig. S1 Association between genome size and gene/TE content. Genome sizes (Mb) were plotted against genes or TE coverage for each Pucciniomycotina species. The lines represent the linear regression. [file 12864_2025_11726_MOESM1_ESM.pdf]

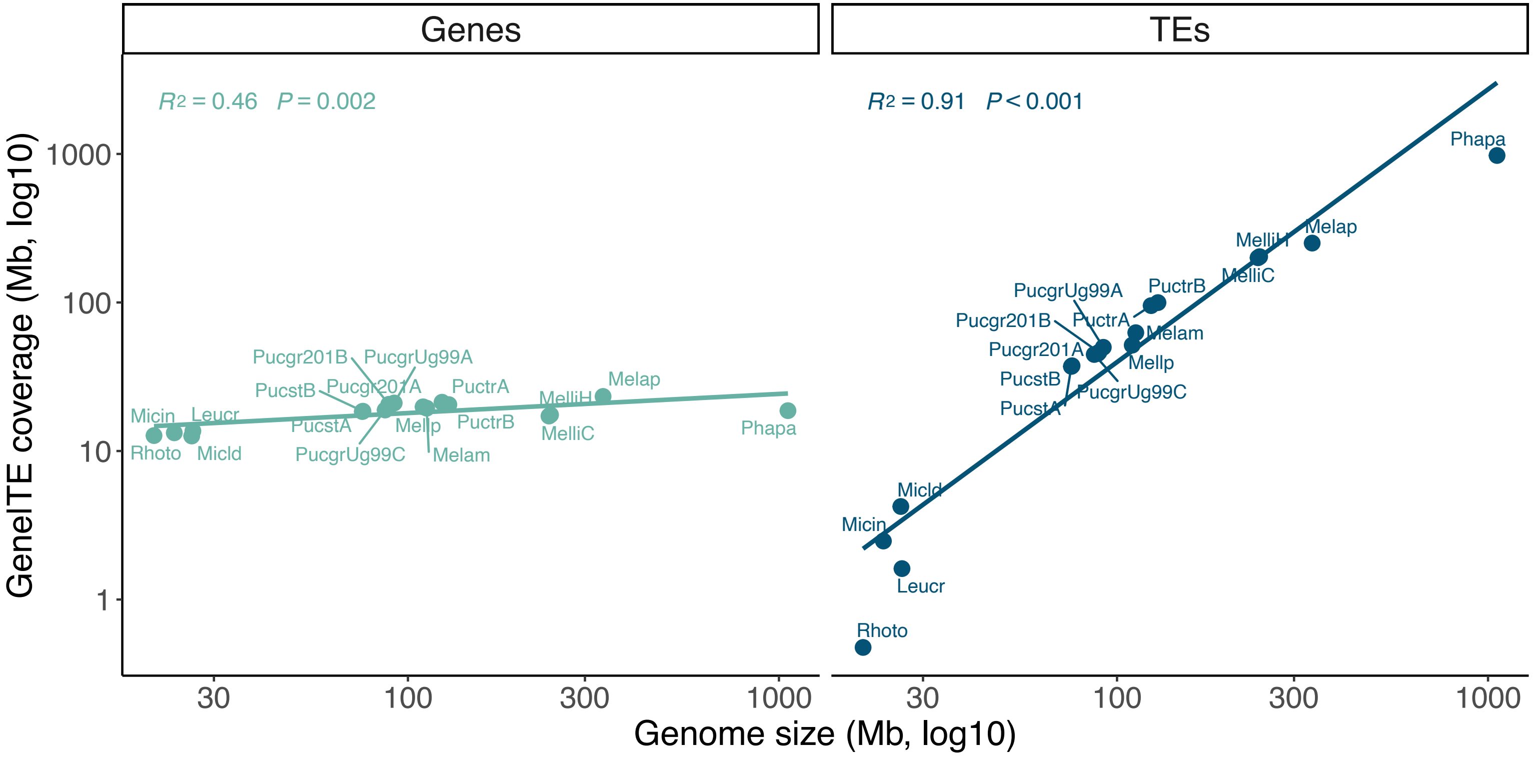

Fig. S1: Association between genome size and gene/TE content. Genome sizes (Mb) were plotted against genes or TE coverage for each Pucciniomycotina species. The lines represent the linear regression.
