# Supplementary material for: Ancestral and recent bursts of transposition shaped the massive genomes of plant pathogenic rust fungi
Source: BMC Genomics. 2025 Jul 1;26:627. doi: 10.1186/s12864-025-11726-3 (PMC12210899; doi:10.1186/s12864-025-11726-3)
Supplement: Supplementary file 2 — Supplementary Material 2: Fig. S2 Improvement of TE annotations through manual curation. The proportion of annotated TE families after REPET detection and annotation (RawR), after the first round of curation (Postpct), after the second round of curation with MCHelper (postMC), after a third round of curation with Pucciniomycotina-guided clustering (PostCDhit). [file 12864_2025_11726_MOESM2_ESM.pdf]

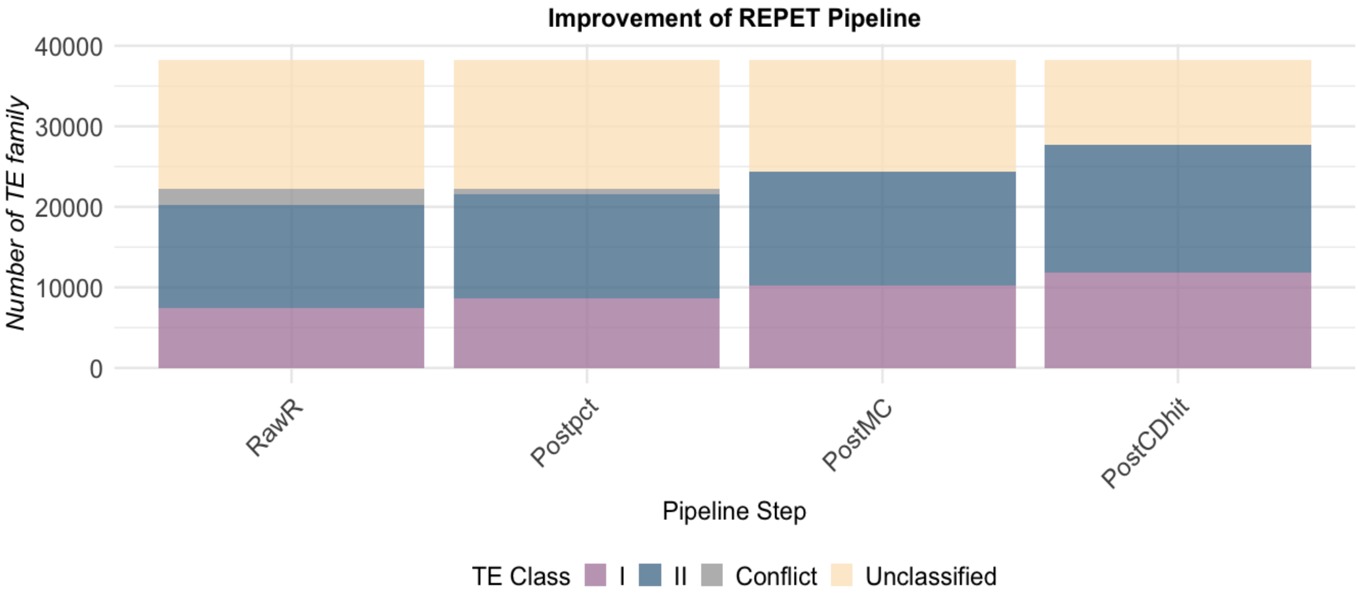

Fig. S2: Improvement of TE annotations through manual curation. The proportion of annotated TE families after REPET detection and annotation (RawR), after the first round of curation (Postpct), after the second round of curation with MCHelper (postMC), after a third round of curation with Pucciniomycotina-guided clustering (PostCDhit).
