# Supplementary material for: Ancestral and recent bursts of transposition shaped the massive genomes of plant pathogenic rust fungi
Source: BMC Genomics. 2025 Jul 1;26:627. doi: 10.1186/s12864-025-11726-3 (PMC12210899; doi:10.1186/s12864-025-11726-3)
Supplement: Supplementary file 3 — Supplementary Material 3: Fig. S3 Shared and specific TE families between the Pucciniomycotina species. Numbers of TE families shared among the 14 genomes based on CDHIT clustering. The bars on the left represent the total number of genes per species. The clusters shared among Pucciniaceae are colored in blue and the clusters shared among Melampsoraceae are colored in red. [file 12864_2025_11726_MOESM3_ESM.pdf]

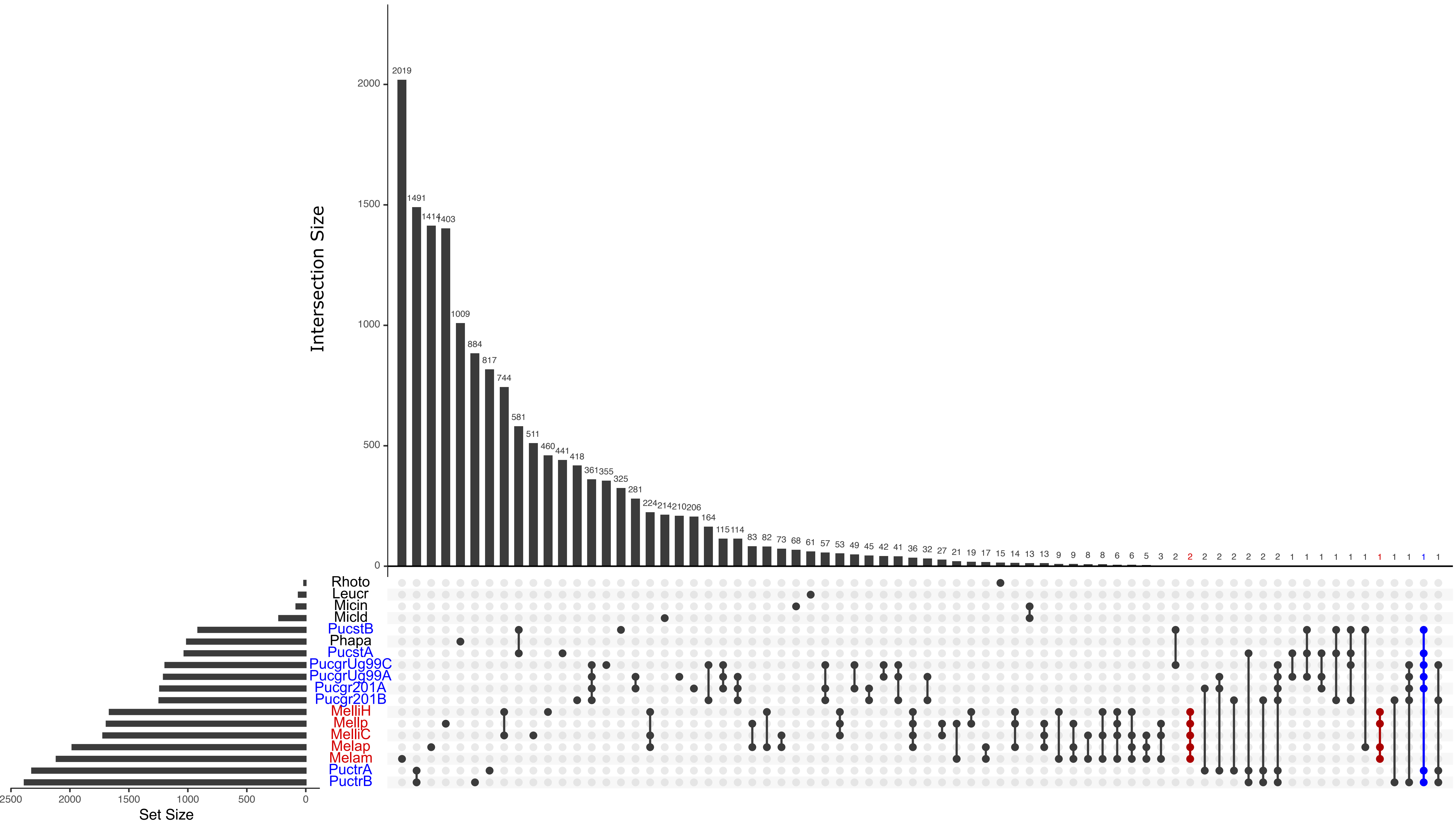

Fig. S3: Shared and specific TE families between the Pucciniomycotina species. Numbers of TE families shared among the 14 genomes based on CDHIT clustering. The bars on the left represent the total number of genes per species. The clusters shared among Pucciniaceae are colored in blue and the clusters shared among Melampsoraceae are colored in red.
