# Supplementary material for: Ancestral and recent bursts of transposition shaped the massive genomes of plant pathogenic rust fungi
Source: BMC Genomics. 2025 Jul 1;26:627. doi: 10.1186/s12864-025-11726-3 (PMC12210899; doi:10.1186/s12864-025-11726-3)
Supplement: Supplementary file 4 — Supplementary Material 4: Fig. S4 Number of LTR-Gypsy elements accumulated through time (million years ago—Mya), based on insertion age for each genome. [file 12864_2025_11726_MOESM4_ESM.pdf]

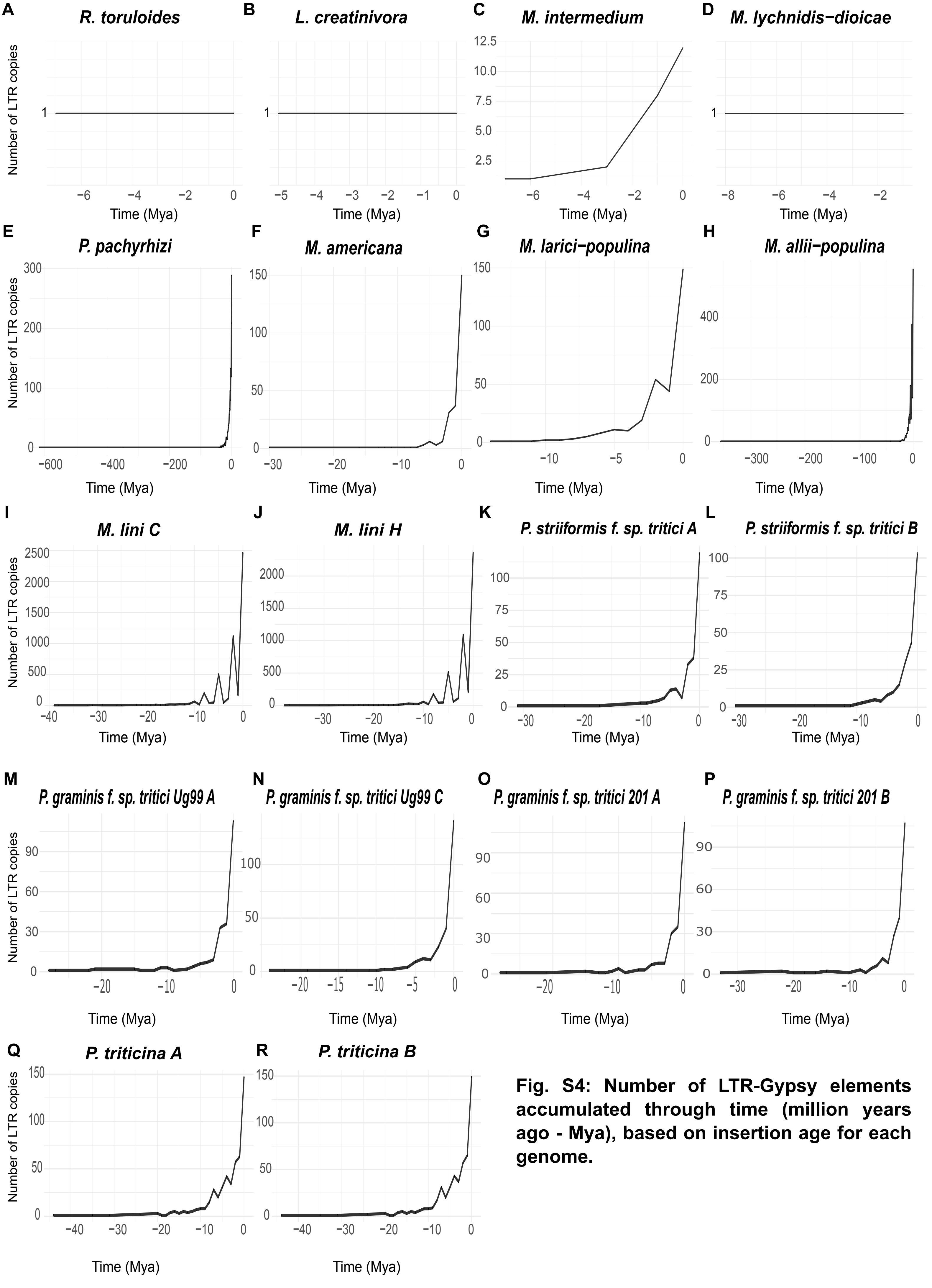

Fig. S4: Number of LTR-Gypsy elements accumulated through time (million years ago - Mya), based on insertion age for each genome.
