# Supplementary material for: Ancestral and recent bursts of transposition shaped the massive genomes of plant pathogenic rust fungi
Source: BMC Genomics. 2025 Jul 1;26:627. doi: 10.1186/s12864-025-11726-3 (PMC12210899; doi:10.1186/s12864-025-11726-3)
Supplement: Supplementary file 5 — Supplementary Material 5: Fig. S5 TE controls involved in methylations are detected in all species. The number of genes involved in TE control corresponds to DNMT1 and DNMT5. [file 12864_2025_11726_MOESM5_ESM.pdf]

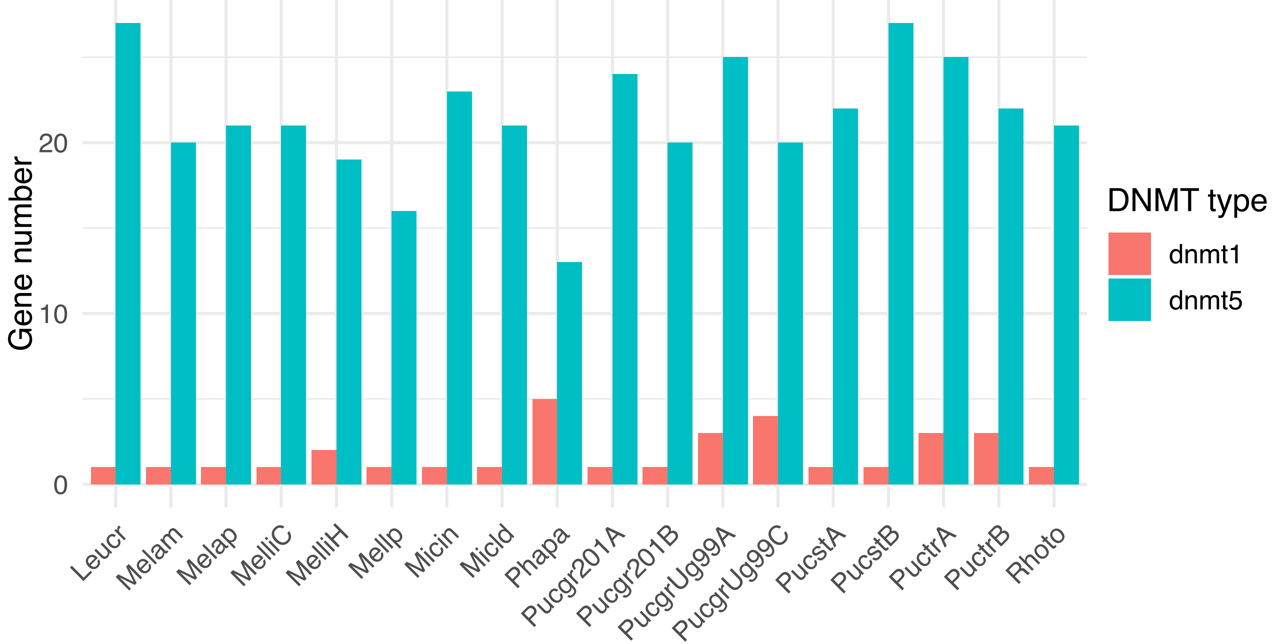

Fig. S5: TE controls involved in methylations are detected in all species. The number of genes involved in TE control corresponds to DNMT1 and DNMT5.
