# Supplementary material for: Ancestral and recent bursts of transposition shaped the massive genomes of plant pathogenic rust fungi
Source: BMC Genomics. 2025 Jul 1;26:627. doi: 10.1186/s12864-025-11726-3 (PMC12210899; doi:10.1186/s12864-025-11726-3)
Supplement: Supplementary file 6 — Supplementary Material 6: Fig. S6 TE controls involved in RNA-induced silencing complex are detected for all genomes. The number of genes involved in TE control corresponds to the RNAi pathway: Dicer-like (DCL; DCR), RNA-dependent RNA polymerase (RDP; RNT; RPAB; RPB), Argonaute (AGO; ARB) for each genome. [file 12864_2025_11726_MOESM6_ESM.pdf]

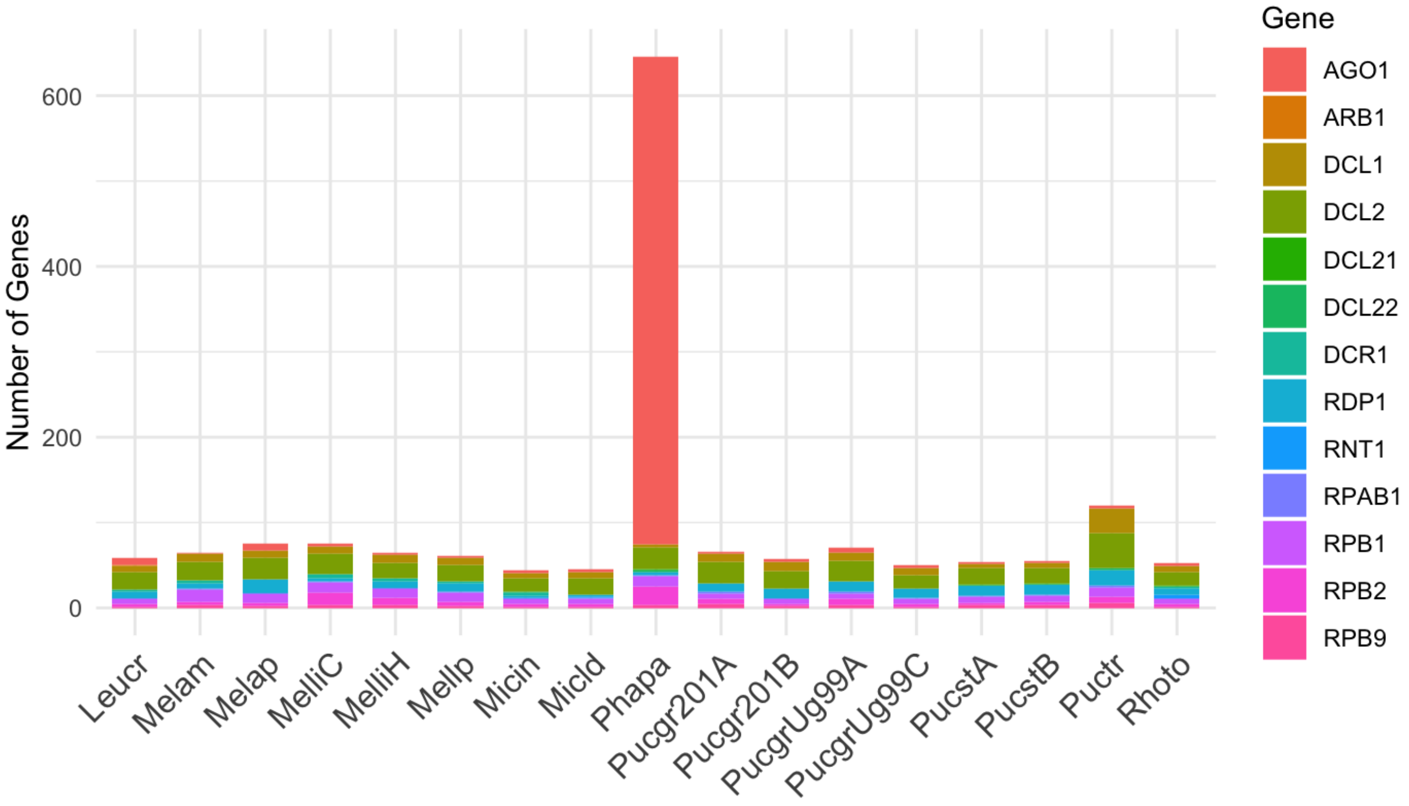

Fig. S6: TE controls involved in RNA-induced silencing complex are detected for all genomes. The number of genes involved in TE control corresponds to the RNAi pathway: Dicer-like (DCL; DCR), RNA-dependent RNA polymerase (RDP; RNT; RPAB; RPB), Argonaute (AGO; ARB) for each genome.
