# Supplementary material for: Ancestral and recent bursts of transposition shaped the massive genomes of plant pathogenic rust fungi
Source: BMC Genomics. 2025 Jul 1;26:627. doi: 10.1186/s12864-025-11726-3 (PMC12210899; doi:10.1186/s12864-025-11726-3)
Supplement: Supplementary file 7 — Supplementary Material 7: Fig. S7 Contraction and Expansion of multigene families in Pucciniomycotina. The phylogenetic tree of the selected Pucciniomycotina species based on single-copy orthogroups from Orthofinder v2.4.0 and infer with Raxml (500 bootstraps) and visualized using iTOL, rooted with Ustilago maydis. Numbers in purple correspond to the number of orthogroups in contractions at each branch or node and yellow at expansions. [file 12864_2025_11726_MOESM7_ESM.pdf]

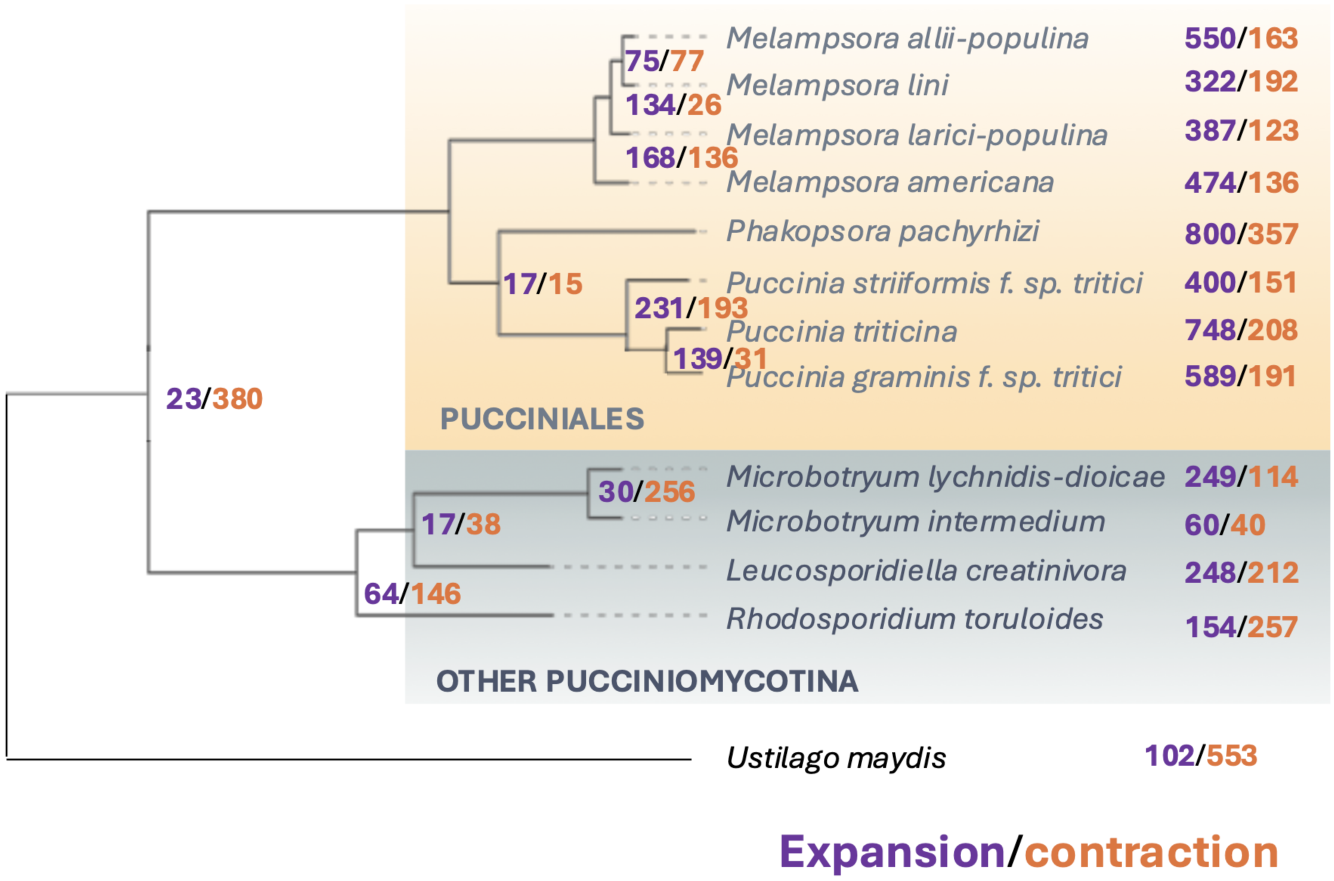

Fig. S7: Contraction and Expansion of multigene families in Pucciniomycotina.
The phylogenetic tree of the selected Pucciniomycotina species based on single-copy orthogroups from Orthofinder v2.4.0 and infer with Raxml (500 bootstraps) and visualized using iTOL, rooted with Ustilago maydis. Numbers in purple correspond to the number of orthogroups in contractions at each branch or node and yellow at expansions.
