# Supplementary material for: Ancestral and recent bursts of transposition shaped the massive genomes of plant pathogenic rust fungi
Source: BMC Genomics. 2025 Jul 1;26:627. doi: 10.1186/s12864-025-11726-3 (PMC12210899; doi:10.1186/s12864-025-11726-3)
Supplement: Supplementary file 8 — Supplementary Material 8: Fig. S8 Contraction of Multigene Families in Pucciniales. Functional annotation of multigene families in contraction in Pucciniales. Genes are re-annotated by EggNOG-mapper v2.1.12. A threshold is set to kept only species with at least 20 proteins per COG category. COG categories correspond to: “- “: no hit; B: chromatin structure and dynamics; C: energy production and conversion; E: amino acid transport and metabolism; G: carbohydrate transport and metabolism; I: lipid transport and metabolism; K: transcription; O: posttranslational modification, protein turnover, chaperones, P: inorganic ion transport and metabolism; Q: secondary metabolites biosynthesis, transport and catabolism; S: function unknown; T: signal transduction mechanisms. [file 12864_2025_11726_MOESM8_ESM.pdf]

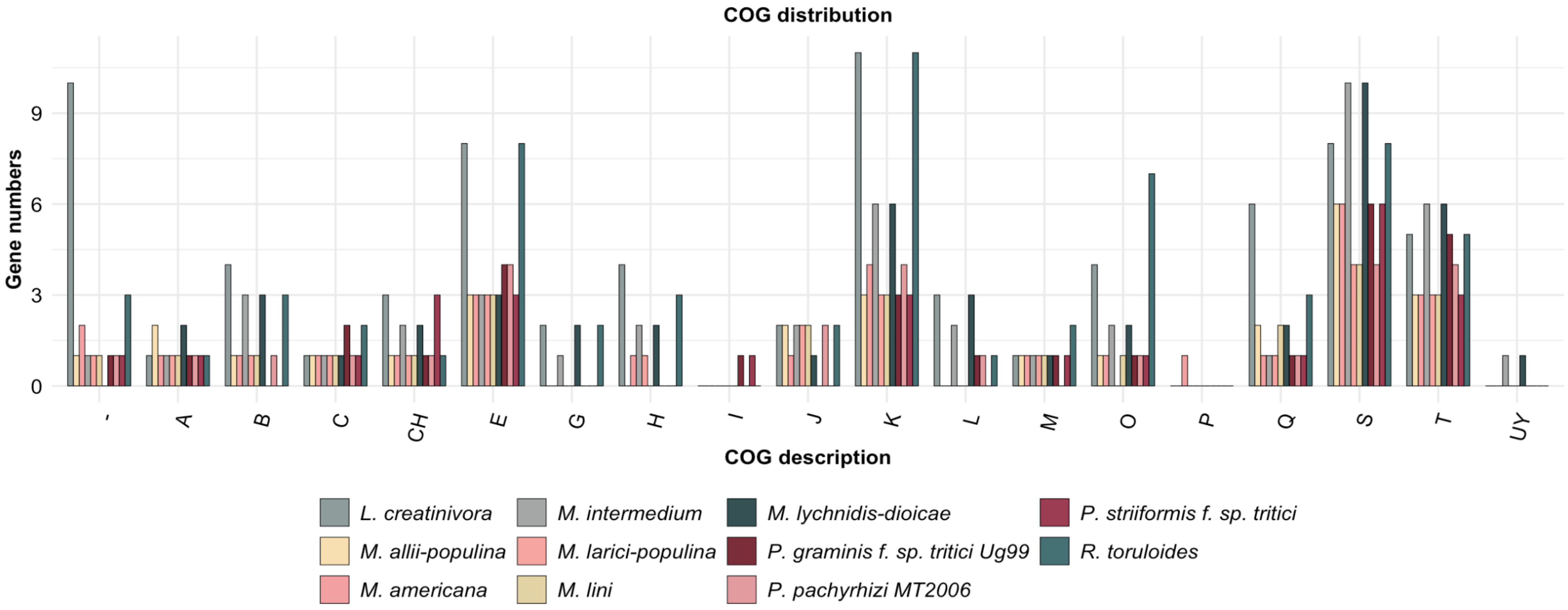

Fig. S8: Contraction of Multigene Families in Pucciniales. Functional annotation of multigene families in contraction in Pucciniales. Genes are re-annotated by EggNOG-mapper v2.1.12. A threshold is set to kept only species with at least 20 proteins per COG category. COG categories correspond to: “-“: no hit; B: chromatin structure and dynamics; C: energy production and conversion; E: amino acid transport and metabolism; G: carbohydrate transport and metabolism; I: lipid transport and metabolism; K: transcription; O: posttranslational modification, protein turnover, chaperones, P: inorganic ion transport and metabolism; Q: secondary metabolites biosynthesis, transport and catabolism; S: function unknown; T: signal transduction mechanisms.
