# Supplementary material for: Ancestral and recent bursts of transposition shaped the massive genomes of plant pathogenic rust fungi
Source: BMC Genomics. 2025 Jul 1;26:627. doi: 10.1186/s12864-025-11726-3 (PMC12210899; doi:10.1186/s12864-025-11726-3)
Supplement: Supplementary file 9 — Supplementary Material 9: Fig. S9 Significant contraction and expansion of multigene families in Pucciniomycotina. A) The phylogenetic tree of the selected Pucciniomycotina species based on a single-copy orthogroup from Orthofinder v2.4.0 and visualized using iTOL, rooted with Ustilago maydis. Numbers in black correspond to the number of significant orthogroups in expansion/contraction at each branch or node. B) COG category annotation of genes involved in expansion and contraction between Pucciniales and other Pucciniomycotina. Contraction and expansion are detected with CAFE v5.0 and orthogroups with a p-value (0.05) are kept. Genes are re-annotated by EggNOG-mapper v2.1.12. The number of genes per species and categories. COG categories functions correspond to: A: RNA processing and modification; C: energy production and conversion; D: cell cycle control, cell division chromosome partitioning; G: carbohydrate transport and metabolism; I: lipid transport and metabolism; K: transcription; L: replication recombination and repair; M: cell wall, membrane, envelope biogenesis; N: cell motility; O: posttranslational modification, protein turnover, chaperones, Q: secondary metabolites biosynthesis, transport and catabolism; S: function unknown; T: signal transduction mechanisms. [file 12864_2025_11726_MOESM9_ESM.pdf]

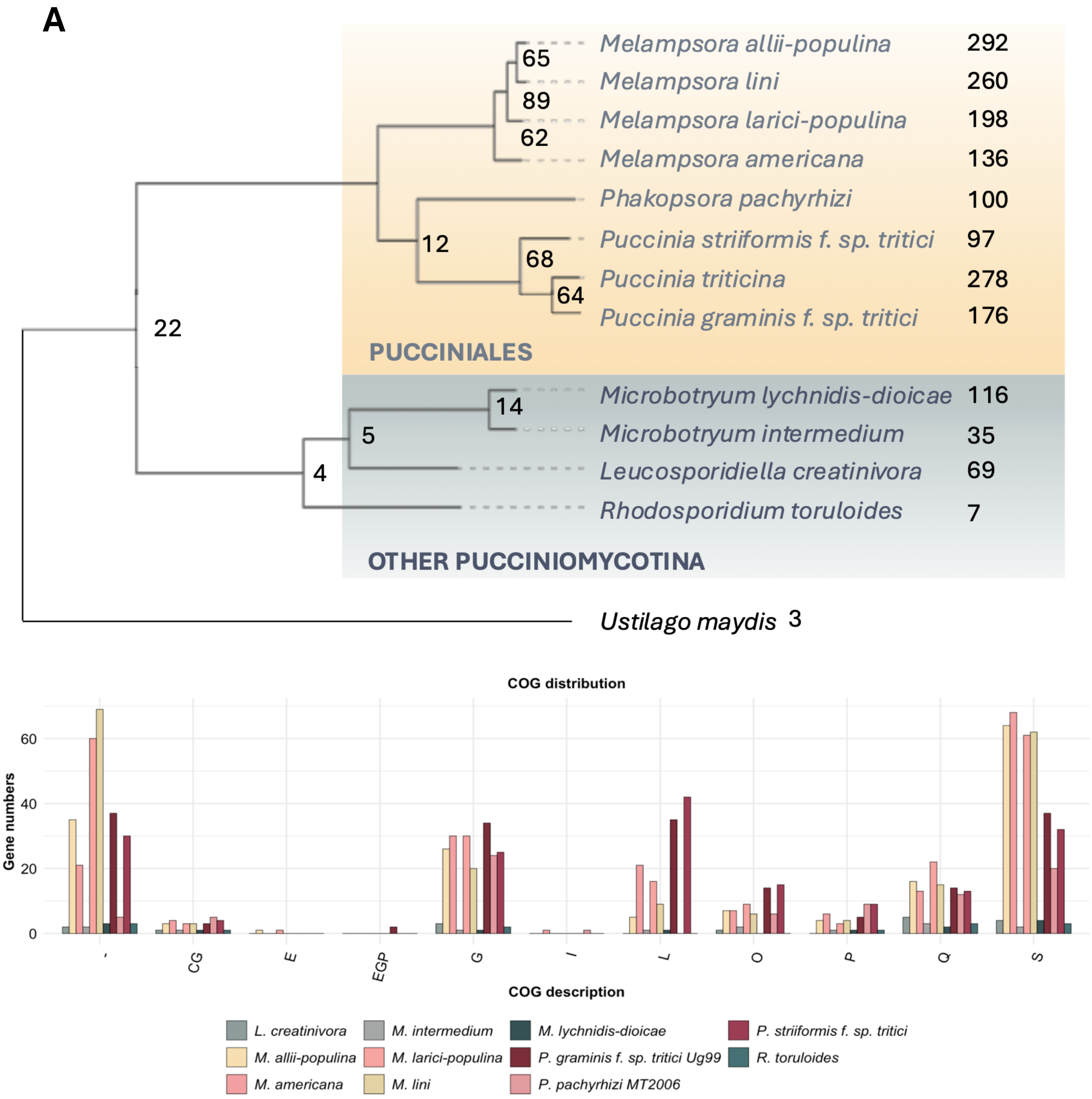

Fig. S9: Significant contraction and expansion of multigene families in Pucciniomycotina. A) The phylogenetic tree of the selected Pucciniomycotina species based on a single-copy orthogroup from Orthofinder v2.4.0 and visualized using iTOL, rooted with Ustilago maydis. Numbers in black correspond to the number of significant orthogroups in expansion/contraction at each branch or node. B) COG category annotation of genes involved in expansion and contraction between Pucciniales and other Pucciniomycotina. Contraction and expansion are detected with CAFE v5.0 and orthogroups with a p-value (0.05) are kept. Genes are re-annotated by EggNOG-mapper v2.1.12. The number of genes per species and categories. COG categories functions correspond to : A: RNA processing and modification; C: energy production and conversion; D: cell cycle control, cell division chromosome partitioning; G: carbohydrate transport and metabolism; I: lipid transport and metabolism; K: transcription; L: replication recombination and repair; M: cell wall, membrane, envelope biogenesis; N: cell motility; O: posttranslational modification, protein turnover, chaperones, Q: secondary metabolites biosynthesis, transport and catabolism; S: function unknown; T: signal transduction mechanisms.
